# Validation of the Structured Interview for the Assessment of Expressed Emotion (E5) in a Sample of Adolescents and Young Adults From the General Population

**DOI:** 10.3389/fpsyg.2021.723323

**Published:** 2021-08-27

**Authors:** Jose-Antonio Muela-Martinez, Lourdes Espinosa-Fernandez, Luis-Joaquin Garcia-Lopez, Maria-Eva Martin-Puga

**Affiliations:** Department of Psychology, University of Jaen, Jaen, Spain

**Keywords:** expressed emotion, adolescence, young adults, parent, measurement

## Abstract

Expressed emotion (EE) is an index of significant others’ attitudes, feelings, and behavior toward an identified patient. EE was originally conceptualized as a dichotomous summary index. Thus, a family member is rated low or high on how much criticism, hostility, and emotional overinvolvement (EOI) s/he expresses toward an identified patient. However, the lack of brief, valid measures is a drawback to assess EE. To cover this gap, the E5 was designed. The objective of this study is to provide psychometric properties of a recent measured in adolescents to be used to tap perceived high levels of EE. The sample was composed by 2,905 adolescents aged from 11–19years; 57% girls. Results demonstrate good factor structure, reliability, construct validity and invariance across gender and age revealed a good fit. As a result, E5 is a brief, valid and reliable measure for assessing expressed emotion in parents of adolescent children.

## Introduction

Expressed emotion (EE) is an indicator of family emotional climate, which carries a high predictive value in the prognosis of different disorders associated with stress and anxiety over the course of their development. This construct accounts for how family members interact with the relative who suffers from a disorder. The EE construct came to the fore during the 1970 and 1980s, when the course of schizophrenia received research attention (Amaresha and Venkatasubramanian, 2012). However, EE has also been found to be related to a worse disease course across different disorders (Muela and Godoy, 2003; Hooley, 2007; Przeworski et al., 2012; Miklowitz et al., 2013; Iles et al., 2014; Ma et al., 2021). What is more, EE has shown to have a varying impact on treatment outcomes. Specifically, Garcia-Lopez et al. (2009) found that high parental EE levels adversely affected the positive treatment outcomes of adolescents with social anxiety. Later, Garcia-Lopez et al. (2014) reported that parent training to reduce EE in a treatment program designed to tackle adolescent social anxiety had a positive effect on the child’s SA improvement, particularly when the EE status of parents shifted from high- to low-EE following treatment.

Expressed emotion is dichotomized into high- and low-EE. Thus, a family with high-EE is one which includes a family member who demonstrates some – or all – of the following characteristics toward the affected relative:

1. Criticism: Dissatisfaction, resentment, and disapproval regarding the affected relative’s behavior.
2. Hostility: Actively excluding or avoiding said person (hostile rejection) or holding a negative view of the person as a whole (generalized hostility).
3. Emotional overinvolvement:
  3.1 Hopelessness: Firmly claiming that there is no solution or chance of things improving in the affected relative’s condition or behavior.
  3.2 Self-sacrifice: Emphasizing the impact that the affected relative’s disorder is having on the family itself.
  3.3 Overprotection: Making excuses for the affected relative and taking on their obligations and responsibilities.
  3.4 Intense emotional displays: Uncontrollable crying, outbursts of anger, etc.

The Camberwell Family Interview (CFI) developed by Vaughn and Leff (1976) was the first instrument to measure EE; today it is still considered the gold standard test for assessing this construct (Masaaki et al., 2004). However, this is a lengthy interview (between 1.5 and 2h), which requires training in order to administer and evaluate it, and which also needs to be corrected for inter-rater reliability. The Five Minute Speech Sample (FMSS; Magana et al., 1986) emerged as an alternative to the CFI. This brief measure requires little training to administer (at least in relation to the CFI) and includes some criterion validity data deemed more than adequate compared with the CFI (Magana et al., 1986; Leeb et al., 1991). However, this still poses the problem of having to perform inter-rater reliability correction, thus requiring the appropriate training.

A number of self-report measures have been developed to assess EE. These include the Level of Expressed Emotion Scale (LEE; Cole and Kazarian, 1988), the Expressed Emotion Adjective Checklist (EEAC; Friedman and Goldstein, 1993), and the Questionnaire Assessment of Expressed Emotion (QAEE; Docherty et al., 1990). However, these measures do not present conclusive data that correlate with the CFI or the FMSS.

Notwithstanding, the Perceived Criticism Scale (PCS; Hooley and Teasdale, 1989), according to its authors, demonstrates strong concurrent validity with the complete CFI, although not with the CFI’s criticism/hostility component (to be expected); this suggests that emotional overinvolvement is not completely independent from criticism.

A more recent self-report measure, namely the Brief Dyadic Scale of Expressed Emotion (Escala Diádica Breve de Emoción Expresada/BDSEE; Medina-Pradas et al., 2011), has yielded good levels of construct reliability and validity as well as statistically significant correlations with the CFI. Both the BDSEE and the PCS have versions that measure not only EE displayed by the affected relative, but also the participants’ perceived EE; the CFI and FMSS only measure the former.

However, all of these alternative measures are still considered too long for us to see a generalized use of them in the clinical setting (Van Humbeeck et al., 2002).

Thus, the aim of this research is to validate an instrument that measures familial EE in a quick and easy manner from the perspective of not only the relative displaying EE, but also of the assessee themselves, and which is applicable to female and male adolescents and young adults.

## Materials and Methods

### Participants

The sample comprised 3,284 students, aged 11–18years, from across every secondary school (IES) in Jaén, a medium-sized city in south-central Spain. Among this sample, 379 participants were excluded from the analyses for not responding to some scale items, thus resulting in missing values. This brought the final sample down to 2,905 participants. Reliability and validity analyses, as well as an E5 exploratory factor analysis, were performed on a sub-sample made up of 580 participants (38.4% male, aged 11–19years: *M*=14.61; *SD*=1.87), whereas a confirmatory factor analysis (CFA) and all other validation analyses were conducted on a sub-sample comprised of the remaining 2,325 participants (47.3% male, 11–19years: *M*=14.32; *SD*=1.62).

### Measures

– Structured Interview for the Assessment of Expressed Emotion: Child version; E5cv (Entrevista Estructurada para la Evaluación de la Emoción Expresada Versión Hijos; E5-vh). A seven-item structured interview with five response options, ranging from 1=“never” to 5=“always” was developed for the purpose of this study. Each item covers a dimension of EE: criticism, generalized hostility, hostile rejection, hopelessness, self-sacrifice, overprotection, and intense emotional displays. However, the reliability and validity analyses rendered the last two items redundant; they were removed for being identified as parental responses when faced with potentially conflictual parent–child situations. In this case, and because a broad sample of people were subject to assessment, the interview format was deemed unfeasible. Given that a structured interview using preset response options is the equivalent to a self-report measure, we opted for a self-rating scale with the following introductory text: “Listed below are some common ways of responding, feeling, and thinking when faced with stressful or confrontational situations. Please put an X in the box against each response, ranging from ‘Never’ to ‘Always,’ rating the frequency with which your mother and/or father reacts in a particular way when a confrontational situation that causes stress, or which may lead to arguments, arises in the home.” Administration time is approximately 5min. The Cronbach’s alpha in this study for the final five-item E5 was 0.81.
– Brief Dyadic Scale of Expressed Emotion (Escala Diádica Breve de Emoción Expresada; Medina-Pradas et al., 2011). This instrument evaluates EE by capturing the view of both members of the dyad; in other words, taking into account the point of view of the parents and children separately. In this study, only the child version was administered. It comprises 14 items scored on a 10-point Likert scale with the following prompts: 1: no or never; 5: regularly or sometimes; and 10: A lot or always. The EE components “criticism” and “emotional overinvolvement” are measured. The Cronbach’s alphas in this study for each component were 0.71 and 0.68, respectively.
– Perceived Criticism Scale (Hooley and Teasdale, 1989). This measure includes four questions designed to assess (a) the degree of perceived criticism the child feels toward their parents and (b) their own self-criticism about their parents’ opinions of them and the degree of anger felt as a reaction to these criticisms. Participants are asked to respond on a scale of 1 (not at all critical/angry) to 10 (very critical/angry). The Cronbach’s alpha in this study for this measure was 0.72. Given that the response scale was the same as for the previous instrument, and that the first item featured in both measures, they were combined to make a 17-item self-report questionnaire.

### Procedure

The sample was recruited from 17 (88% state-run) secondary schools (IES) in Jaén province located across rural (18%) and urban (82%) towns. Following the signing of an agreement between the University of Jaén and the Department of Education of the Andalusian Regional Government (IES are legally dependent on this authority), all schools throughout the province were informed about the study objectives and their collaboration was requested. Those secondary schools which agreed to take part received a visit from the assessment team; after explaining to the students the methodology and study aims, the potential participants were given a consent form which they could return with their parents’ signatures if they wished to proceed. Subsequently, the different assessment tools were administered.

First, only the seven-item E5 interview was administered; this was done until a minimum of 580 respondents had completed the questionnaire in its entirety. This followed the recommendations made by Hogarty et al. (2005), whereby a target sample size of at least 500 would provide precise enough estimates with Exploratory Factor Analysis under the least favorable conditions (for example, low communalities or three items per factor, which were deemed possible owing to the uncertainty of whether this was the case or not). This tool was administered in one or two classrooms for each school at the start of the term (selected at random) until the desired sample was reached. The interview took 5min to complete and was carried out as a group activity during class time.

After analyzing the results, the assessment team moved onto the counterbalanced administration of all measures (five-item E5, BDSEE, and PCS) across all remaining year groups and classes. Similarly, administration time was approximately 15min.

## Results

The analysis was carried out using R (version 4.0.2; R Core Team, 2020). First, the Cronbach’s alpha for the seven-item E5 on the 580-participant sample was calculated. A result of 0.763 was obtained. Item 6 [“(my father/my mother) ends up taking responsibility for what should fall to me”] and item 7 [“(my father/my mother) feels so worried and sad that she/he can hardly refrain from crying”] did not contribute to the scale’s internal consistency; they surpassed it and remained the same, respectively. Participants found both items to be ambiguous. For these reasons, the decision was taken to remove the two items from the instrument. Thus, the Cronbach’s alpha for the five-item E5 was 0.792. Neither alpha value increased when each item was eliminated, nor the correlations between each item and the total scale fluctuated between 0.50 and 0.63.

An exploratory factor analysis was carried out with extraction by principal axis factoring (PAF). The results obtained with Bartlett’s test [*χ*^2^(10, *N*=580)=781.88; *p*<0.001] and the Kaiser-Meyer-Olkin (KMO) test (KMO=0.811), indicated data adequacy for the factor analysis. The obtained factor score explained 43.95% of the total variance (Table 1).

**Table 1:** Exploratory factor analysis.

Items	Factor 1
1. Criticism	0.65
2. Generalized hostility	0.68
3. Hostile rejection	0.74
4. Hopelessness	0.68
5. Self-sacrifice	0.56

The following analyses were performed on the entire sample comprising 2,325 participants.

The descriptive statistics of the E5 are shown in Table 2.

**Table 2:** Descriptive statistics of the E5 items and confirmatory factor analysis (CFA) standardized factor loadings.

Items	*M*	*SD*	Skewness	Kurtosis	Factor loadings
1	2.25	1.07	0.51	−0.47	0.60
2	1.56	0.83	1.51	2.06	0.71
3	1.54	0.82	1.53	1.89	0.71
4	1.43	0.80	2.01	3.90	0.74
5	1.45	0.78	1.85	3.22	0.62

### Confirmatory Factor Analysis

Confirmatory factor analysis was used to verify the factor structure of the E5 by adjusting the model. Taking into account the items’ ordered response categories, the weighted least squares mean and variance adjusted (WLSMV) robust estimator was used. Values close to 0.95 for CFI and Tucker–Lewis index (TLI), 0.06 for scaled root mean square error of approximation (RMSEA), and 0.08 for standardized root mean square residual (SRMR) indicated a relatively good fit for the model (Hu and Bentler, 1999).

The results indicated a good fit for the data [*χ*^2^(5)=24.487; *p*<0.001; CFI=0.994; TLI=0.988; RMSEA=0.041 (0.026, 0.058); SRMR=0.021].

The model’s factor loadings, with values between 0.60 and 0.74, are shown in Table 2.

### Measurement Invariance

The WLSMV robust estimator with theta parameterization was used to test the model’s measurement invariance (MI) by sex and age. The procedure proposed by Wu and Estabrook (2016) was followed to identify models with ordered categorical variables. Chi-square difference testing was used to compare the increasingly restrictive models. However, change in CFI (ΔCFI)>0.01 and change in RMSEA (ΔRMSEA)>0.015 were used as criteria for rejecting measurement invariance (Chen, 2007), considering the sensitivity of the likelihood ratio tests *χ*^2^ to sample size.

Regarding invariance across sexes, the configural invariance model yielded acceptable fit (see Table 3 for the fit of the tested invariance models). Threshold invariance was met by constraining item thresholds to be equal across all groups, indicated by a non-statistically significant Δ*χ*^2^ (*p*=0.476). Similarly, loading invariance was supported by constraining loadings and thresholds to be equal for boys and girls (*p*=0.856). All the invariance models subject to testing demonstrated a good fit.

**Table 3:** Model fit statistics for evaluating measurement invariance (MI) across sex and age.

Model	*χ* ^2^	*df*	CFI	TLI	RMSEA [90% CI]	SRMR	∆*χ*^2^	*df*	∆CFI	∆RMSEA
Sex										
Configural	28.401^**^	10	0.994	0.989	0.040 [0.023, 0.057]	0.023				
Threshold	40.713^**^	20	0.994	0.994	0.030 [0.016, 0.043]	0.023	9.60	10	0.000	0.010
Loading	35.760	24	0.996	0.997	0.021 [0.000, 0.034]	0.024	1.33	4	−0.002	0.009
Age										
Configural	32.599^***^	10	0.993	0.986	0.044 [0.028, 0.062]	0.025				
Threshold	56.456^***^	20	0.988	0.988	0.040 [0.028, 0.052]	0.025	19.01^*^	10	0.005	0.004
Loading	48.648^**^	24	0.992	0.993	0.030 [0.018, 0.042]	0.025	1.24	4	−0.004	0.010

*χ*^2^, Satorra–Bentler scaled chi-square test; df, degrees of freedom; CFI, scaled comparative fit index; TLI, scaled Tucker–Lewis index; RMSEA, scaled root mean square error of approximation; 90% CI, 90% confidence interval around RMSEA; SRMR, standardized root mean square residual; ∆*χ*^2^, change in *χ*^2^; ΔCFI, change in CFI; and ΔRMSEA, change in RMSEA.

* *p*<0.05;

** *p*<0.01;

*** *p*<0.001.

Two groups were formed in order to analyze invariance by age: 11–14years (Group 1) and 15–19years (Group 2). The configural invariance model yielded a good fit (see Table 3). Threshold and loading invariances were also met (*p*=0.040 and *p*=0.871, respectively). Although Δ*χ*^2^ was statistically significant for threshold invariance, the increments in CFI and RMSEA fell below the established cut-off points (ΔCFI=0.005; ΔRMSEA=0.004). As shown in Table 3, the fit was good for all examined invariance models.

### Reliability and Validity

Once again, the scale’s internal consistency was measured using Cronbach’s alpha. The value obtained was 0.81.

In order to measure convergent validity, the correlation coefficients between the E5 with the BDSEE criticism and overinvolvement subscales, and the PCS, were calculated. Large or moderate correlations were observed with the CC and SIP subscales of the BDSEE (0.54 and 0.47, respectively), as well as with the PCS (0.47).

## Discussion

The aim of this research was to develop a brief, valid, and reliable measure for assessing expressed emotion in parents of adolescent children. The results obtained show that this objective had been achieved, reinforced by the fact that the final sample was sizable and that the invariance models by sex and age revealed a good fit. This allowed us to compare expressed emotion between boys and boys as well as between younger and older participants.

As previously mentioned, EE is a hugely important variable when it comes to studying the course of numerous disorders. However, the two best available instruments for measuring EE (Hooley, 2007), namely the CFI (Vaughn and Leff, 1976) and the FMSS (Gottschalk et al., 1988), present a series of disadvantages that place restrictions on their practical use (especially the need to train the interviewer how to assess and correct the instruments, which calls for several evaluators with high interrater reliability). In terms of the measure used for our study, the E5-vh (child version), interviewees were asked a general question about situations that potentially generate EE and were given a list of possible responses so participants could decide how often their parents reply this way. As observed, the evaluator requires no training in administering, correcting, and interpreting the responses, given that they have already been codified. This gives the E5-vh an advantage over the two most widely used, conventional instruments for assessing EE: the CFI and the FMSS. Another advantage of the E5-vh is its short administration time (approximately 5min); the hour and a half to 2h needed for the CFI, plus correction time, make it very expensive, whereas the 5min allocated to the FMSS may not be enough (if sufficient training is lacking) to obtain results representative of EE in respondents (Masaaki et al., 2004). The decision to administer a structured interview using preset response options, similar to a self-rating scale, was taken owing to the high number of sample participants; it would not have been possible to assess so many people by conducting one-on-one interviews, despite the measure being brief in nature. It might have been thought more logical to present the instrument as a scale directly rather than as a structured interview with preset responses; however, the authors behind this research drew upon their clinical experience to suggest that if the instrument is intended for use in clinical practice, then there are more possibilities to achieve this using the interview format than by using a scale. Regardless, the equivalence of both formats herein means that any potential measuring differences derived from using one format over the other fall within acceptable levels when compared with the potential benefits.

Being able to assess the perceived EE of the participant instead of just evaluating that demonstrated by the family member (as in the case of the CFI and FMSS), makes this instrument a more interesting alternative. What is more, this approach is consistent with the latest, state-of-the-art methods for assessing EE: for example, the scales used in this study to calculate the E5’s convergent validity: the BDSEE and the PCS. There is a parent version of the E5, which assesses EE exhibited by family members themselves; its validation is currently undergoing study by our research team.

The instrument’s internal consistency was analyzed, yielding some high values. In earlier studies, Cronbach’s alpha increased when two dimensions were removed from the instrument: overprotective behavior and intense emotional displays. It is likely that the items in question were not worded properly or that the characteristics of the selected sample may, in part, have led to these results. This study examines adolescents and young adults from the general population. Expressed emotion has always been studied from the perspective of a disorder – schizophrenia in the early days of EE to social anxiety most recently: Espinosa-Fernandez et al. (2016). However, on this occasion, the general population is considered, leaving things open to situations that may cause EE to surface. Thus, it is not uncommon to see parents’ complaints in everyday situations take on forms referred to in the first five items of the E5. Scolding a child because of their behavior (criticism), discrediting a child (hostility), complaining about how the child is incapable of altering their behavior (hopelessness), and pointing out the efforts that the child’s behavior demands (self-sacrifice) can occur in more or less normal situations. However, intense emotional displays such as uncontrollable crying can more easily be associated with specific situations arising from more problematic contexts, much like those present over the course of a disorder in which EE has always been studied. Thus, intense negative emotions commonly appear and feed into disruptive contexts while also exacerbating the problem into a vicious circle; the more attention one pays to the emotion, the worse it gets, the greater the rumination, and the more situation-based negativity being fed back (Cano and Goubert, 2017; Müller et al., 2019).

Regarding parental overprotection, EE emerges in situations whereby parents take care of the tasks and activities that should fall to their children, which creates the perception among children that their parents do not trust in their capabilities (Akbari et al., 2021). However, in young people without a specific condition which might justify this distrust, any displays of overprotection could be interpreted by the child as their parents’ interfering in their lives. For this age group (average age of 14 in this study sample), the child may perceive this as wrong behavior on the part of their parents, and not as something they do because they do not trust their capabilities. In other words, the child would not see it as a display of EE, which is why this fails to correlate with the complete instrument.

From very early on, Vizcarro and Arévalo (1987) understood EE as a construct made up to two components: criticism (encompassing criticism, generalized hostility, and hostile rejection) and emotional overinvolvement (which would include the other four components mentioned above). Thus, selecting an item for each aspect to create the E5 means that we have, in fact, three items for the criticism component and four items for emotional overinvolvement. Finally, we have ended up with five items, although they all belong to a single factor.

The fact that the E5 shows a single-factor structure suggests that the hopelessness and self-sacrifice items, despite having an emotional overinvolvement component in the sense of exhibiting excessive emotivity, in fact continued to be perceived by the child as their parents’ reproaches. Hence, those items are loaded under the same factor as criticism, generalized hostility, and hostile rejection. This is also coupled with the fact that the correlation indices of the E5 with the BDSEE’s overinvolvement scale are similar to those, which are shown to measure criticism directly (the criticism scale of the BDSEE and PCS).

This study has some limitations. First, the E5 was administered as a self-rating scale and not as a structured interview with preset responses. However, and as commented previously, this type of administration technique was not expected to generate significant bias (the alternative, namely the interview format, was not viable given the high number of assessees). Another limitation may derive from the fact that the authors studied children’s perceived EE and not EE demonstrated by parents, which has been the traditional approach. That said, a new study which includes this variable is currently in the preparation stage; because the new sample comprises parents of adolescent children, analyses can be run which examine the correlation between what parents think they express and what their children perceive. Lastly, a sample with some type of pathology could have been used to examine whether high EE predicts the course of the associated developmental disorder, as expected based on earlier literature. This study is also underway; using the current general sample, the research team is evaluating whether there are any individuals who exhibit an anxiety disorder in order to conduct a follow-up and to test the predictive value of the previously shown EE level.

Despite this, the E5 represents an appealing alternative to all other available EE measures, given its quick and easy administration and correction method. It constitutes a brief, valid, and reliable measure for assessing expressed emotion in parents of adolescent children. Furthermore, its simplicity of use renders it as a useful tool for screening large groups or in personalized clinical practice.

## Data Availability Statement

The raw data supporting the conclusions of this article will be made available by the authors, without undue reservation.

## Ethics Statement

The studies involving human participants were reviewed and approved by UJA-19102. Written informed consent to participate in this study was provided by the participants’ legal guardian/next of kin.

## Author Contributions

J-AM-M contributed to data collection and data analysis, and wrote the manuscript. LE-F coordinated data collection and wrote the manuscript. L-JG-L contributed to data collection and manuscript revision, and wrote part of the results section. M-EM-P performed part of the statistical analysis, wrote part of the results section, and contributed to manuscript revision. All authors contributed to the article and approved the submitted version.

## Conflict of Interest

The authors declare that the research was conducted in the absence of any commercial or financial relationships that could be construed as a potential conflict of interest.

## Publisher’s Note

All claims expressed in this article are solely those of the authors and do not necessarily represent those of their affiliated organizations, or those of the publisher, the editors and the reviewers. Any product that may be evaluated in this article, or claim that may be made by its manufacturer, is not guaranteed or endorsed by the publisher.
